# Applicability of single-step genomic evaluation with a random regression model for reproductive traits in turkeys (*Meleagris gallopavo*)

**DOI:** 10.3389/fgene.2022.923766

**Published:** 2022-08-24

**Authors:** Bayode O. Makanjuola, Emhimad A. Abdalla, Benjamin J. Wood, Christine F. Baes

**Affiliations:** ^1^ Centre for Genomic Improvement of Livestock, Department of Animal Biosciences, University of Guelph, Guelph, ON, Canada; ^2^ School of Veterinary Science, University of Queensland, Gatton, QLD, Australia; ^3^ Hybrid Turkeys, Kitchener, ON, Canada; ^4^ Institute of Genetics, Vetsuisse Faculty, University of Bern, Bern, Switzerland

**Keywords:** pedigree and genomics, random regression analysis, hatchability, fertility, turkeys

## Abstract

Fertility and hatchability are economically important traits due to their effect on poult output coming from the turkey hatchery. Traditionally, fertility is recorded as the number of fertile eggs set in the incubator (FERT), defined at a time point during incubation by the identification of a developing embryo. Hatchability is recorded as either the number of fertile eggs that hatched (hatch of fertile, HOF) or the number hatched from all the eggs set (hatch of set, HOS). These traits are collected throughout the productive life of the bird and are conventionally cumulated, resulting in each bird having a single record per trait. Genetic evaluations of these traits have been estimated using pedigree relationships. However, the longitudinal nature of the traits and the availability of genomic information have renewed interest in using random regression (RR) to capture the differences in repeatedly recorded traits, as well as in the incorporation of genomic relationships. Therefore, the objectives of this study were: 1) to compare the applicability of a RR model with a cumulative model (CUM) using both pedigree and genomic information for genetic evaluation of FERT, HOF, and HOS and 2) to estimate and compare predictability from the models. For this study, a total of 63,935 biweekly FERT, HOF, and HOS records from 7,211 hens mated to 1,524 toms were available for a maternal turkey line. In total, 4,832 animals had genotypic records, and pedigree information on 11,191 animals was available. Estimated heritability from the CUM model using pedigree information was 0.11 ± 0.02, 0.24 ± 0.02, and 0.24 ± 0.02 for FERT, HOF, and HOS, respectively. With random regression using pedigree relationships, heritability estimates were in the range of 0.04–0.09, 0.11–0.17, and 0.09–0.18 for FERT, HOF, and HOS, respectively. The incorporation of genomic information increased the heritability by an average of 28 and 23% for CUM and RR models, respectively. In addition, the incorporation of genomic information caused predictability to increase by approximately 11 and 7% for HOF and HOS, respectively; however, a decrease in predictability of about 12% was observed for FERT. Our findings suggest that RR models using pedigree and genomic relationships simultaneously will achieve a higher predictability than the traditional CUM model.

## Introduction

Turkey meat continues to be a popular meat for consumption with a total production of approximately 6 million tonnes in 2019 (FAO, 2021). The continuous production of turkey poults is dependent on reproductive efficiency. Therefore, improvement of reproductive efficiency increases the production of turkey poults and has a direct effect on the economic growth of the industry. Given the importance of reproductive efficiency in turkeys, research emphasis has focused more on improving egg production (Nestor et al., 1996; Kranis et al., 2007), with less emphasis placed on fertility and hatchability traits. In contrast to egg production, fertility and hatchability traits in turkeys are more directly related to the production of poults. Furthermore, these traits are significant in improving reproductive efficiency in turkeys as they are easily and regularly collected over the productive life of the bird, as well as being influenced by genetic and environmental factors (Wolc and Olori, 2009).

Traditionally, genetic evaluation of these traits has been performed using cumulative (CUM) records collected over the productive life of the bird (Case et al., 2010). However, the longitudinal nature of these traits allows the opportunity to use a model that accounts for the differences in records collected at different time points. For longitudinal traits, random regression (RR) has often been proposed as the model of choice to better account for genetic and environmental variances at different time points. The first potential and practical application of RR was implemented on test-day milk yield in dairy cattle (Schaeffer and Dekkers, 1994). Since its first application, RR has been used to estimate genetic parameters for carcass conformation in beef cattle (Englishby et al., 2016), egg production and body weight in chicken (Anang et al., 2002; Rovadoscki et al., 2016), survival rate in dairy cattle (Sasaki et al., 2015), and body weight in goats (Kheirabadi and Rashidi, 2016). Furthermore, a broiler breeder study by Makanjuola et al. (2021) reported higher genetic gain with the RR model than the cumulative model for hatch of fertile trait.

The availability of genomic information in many species has allowed for a better estimation of the relationships between individuals. The combination of this information with pedigree information simultaneously in a single-step genomic best linear unbiased prediction (ssGBLUP) (Legarra et al., 2009; Misztal et al., 2009) has been shown to outperform the traditional pedigree BLUP approach (Aguilar et al., 2010; Christensen and Lund, 2010). Based on the ssGBLUP approach, Abdalla et al. (2019) observed a 16% increased accuracy for walking score in turkeys over traditional pedigree BLUP. In addition, Emamgholi Begli et al. (2021) observed that accuracies obtained with RR ssGBLUP were generally equal to or higher than those obtained with RR-PBLUP for egg production traits. Similarly, Oliveira et al. (2019) reported higher validation reliabilities for genomic estimated breeding values (GEBV) in comparison to parent averages for milk production traits in dairy cattle when using a multi-trait RR test-day model. Given the benefits of increasing the prediction accuracy with genomic information, as well as the limited number of RR ssGBLUP studies in turkeys, the aims of this study were to 1) estimate genetic parameters for FERT, HOS, and HOF in turkeys using CUM and RR models with pedigree and genomic information and 2) compare the predictive ability of CUM and RR models when using pedigree and genomic information.

## Materials and methods

### Phenotypes and pedigree data

Phenotypic data for this study were provided by Hybrid Turkeys, Kitchener, Canada. In total, 63,935 egg production records collected on a biweekly basis from a purebred turkey female line were available from 2010 to 2019 (Tables 1, 2). These egg production records were collected from 7,211 hens such that there was a total of 7,211 cumulative records, which indicates one record per hen. Cumulative record for each hen was calculated as the total number of eggs produced throughout the productive life of the hen, and this was subsequently used to derive the cumulative fertility and hatchability records. The 7,211 hens were mated to 1,524 toms, and eggs were set in the incubator biweekly throughout the productive life of the hen between 38 and 62 weeks of age. Individual hens were artificially inseminated, and trap-nest collected eggs were labeled with the identity of the hen. Consequently, this provided a pedigree for the progeny, as well as identification for the following hatchery traits. Fertility (FERT) records were collected by a process called candling, whereby light is passed through the eggs to determine the presence of a developing embryo. Hence, FERT was measured as the percent proportion of fertile eggs over the total egg set. Following the collection of fertility records, records of a successful hatching of an egg were used to calculate hatch of set (HOS), which is the proportion of all egg sets that hatched. Finally hatch of fertile (HOF) was defined as the proportion of fertile eggs that successfully hatched. Pedigree data for all animals with phenotypic records were provided and consisted of 15 generations and 11,191 individuals.

**TABLE 1:** Descriptive statistics of the evaluated traits hatch of set (HOS), hatch of fertile (HOF), and fertility of set (FERT) including the number of records for each model, mean, standard deviation, and the number of records in the training and validation populations for each model.

Model	Trait	Mean ± SD	Number of records	Training population	Validation population
**Cumulative (CUM)**	HOS	67.94 ± 16.11			
	HOF	80.86 ± 14.52	7,211	6,447	764
	FERT	81.27 ± 14.02			
**Random regression (RR)**	HOS	68.64 ± 27.20			
	HOF	81.10 ± 26.07	63,935	56,471	7,464
	FERT	82.03 ± 22.75			

**TABLE 2:** Descriptive statistics for hatch of set (HOS), hatch of fertile (HOF), and fertility of set (FERT) including the number of records, mean, standard deviation (SD), and coefficient of variation (CV) for the different time points (38–62 weeks).

Week	Number of records	FERT ± SD	(FERT) CV%	HOF ± SD	(HOF) CV%	HOS ± SD	(HOS) CV%
**38**	2,838	84.06 ± 21.06	25.06	83.57 ± 25.49	30.50	71.53 ± 27.20	38.03
**40**	5,863	83.21 ± 19.47	23.39	83.69 ± 22.25	26.58	70.72 ± 24.70	34.92
**42**	6,395	82.29 ± 19.07	23.18	83.79 ± 21.02	25.08	69.97 ± 23.44	33.50
**44**	5,817	83.70 ± 19.06	22.78	83.80 ± 21.68	25.87	71.20 ± 23.84	33.49
**46**	5,687	84.06 ± 19.66	23.38	82.57 ± 23.21	28.11	70.76 ± 24.71	34.92
**48**	5,649	84.10 ± 20.55	24.44	82.42 ± 23.90	28.99	71.02 ± 25.36	35.71
**50**	5,507	83.84 ± 21.89	26.11	81.87 ± 24.99	30.53	70.56 ± 26.48	37.53
**52**	5,571	81.97 ± 23.84	29.08	81.40 ± 26.28	32.28	69.04 ± 27.74	40.18
**54**	4,781	81.83 ± 24.10	29.45	80.16 ± 27.37	34.14	67.85 ± 28.42	41.88
**56**	4,544	81.19 ± 24.76	30.50	79.82 ± 28.00	35.08	67.48 ± 28.85	42.75
**58**	4,348	79.96 ± 25.71	32.20	77.00 ± 30.25	39.28	64.73 ± 30.11	46.51
**60**	3,709	77.92 ± 27.61	35.43	75.82 ± 31.56	41.62	62.63 ± 31.16	49.75
**62**	3,226	74.48 ± 30.02	40.31	73.55 ± 34.40	46.77	59.33 ± 32.62	54.99

### Genotype data

Of the animals with phenotypic records, a total of 4,832 animals were genotyped using a proprietary 65K SNP panel (Illumina, Inc). The genotype call rate was 94%, and missing genotypes were imputed using AlphaImpute version 2 (Whalen and Hickey, 2020). For imputation, both pedigree and population algorithms were used with a reference population of 1,626 animals. Default settings in AlphaImpute were used for imputation; however, the peeling and phasing cycles were increased to 50 cycles for pedigree and population algorithms, respectively. Increasing the phasing and peeling cycles was performed as a measure to increase the probability of achieving high-confidence-phased haplotypes and correctly calling the genotypes. An error rate of 0.01% was allowed for genotype calls. Imputation accuracy was estimated using allelic *r* ^2^, which is less dependent on allele frequencies and was greater than 98%. For quality control measures, non-autosomal SNP markers and autosomal SNP markers with MAF less than 0.05 and significantly deviating from Hardy–Weinberg equilibrium (P < 1 × 10^−8^) were excluded. After editing, there were a total of 35,751 SNP markers retained for further analysis.

### Statistical analysis

#### Best linear unbiased prediction

To investigate the influence of different parameters on reproductive traits, a CUM animal mixed model using only a single record per animal and a RR animal mixed model using all available records per animal were applied to estimate genetic parameters based only on pedigree relationships.

##### Cumulative model

With the CUM model, the following mixed model equation is used to estimate genetic parameters for reproductive traits:

$$y_{\mathrm{ij}}=\mu +hw_{i}+a_{j}+e_{\mathrm{ij}},$$

where $y_{\mathrm{ij}}$ is a vector of the CUM record of either FERT or HOF or HOS for the *j*th animal belonging to the *i*th hatch week, μ is the overall mean, $hw_{i}$ is a vector of the fixed effect of the *i*th hatch week, and $a_{j}$ is a vector of the random genetic effect of the animal. The assumption of the random effects was: $a_{j}∼N\left(0,A\sigma_{a_{j}}^{2}\right)$ , where $\sigma_{a_{j}}^{2}$ is the additive genetic variance of the animal, $\sigma_{e_{ij}}^{2}$ is the error variance, and A is the numerator relationship matrix.

### Random regression model

For the RR model, the following mixed model equation is used to estimate genetic parameters for reproductive trait biweekly records:

$$y_{\mathrm{ijkno}}=\mu +hw_{i}+\mathrm{eh}w_{j}+\overset{3}{\underset{l=1}{\sum }}b_{l}X_{\mathrm{lk}}+\overset{3}{\underset{l=0}{\sum }}a_{\mathrm{Fnol}}Z_{\mathrm{lk}}+\overset{3}{\underset{l=0}{\sum }}pe_{\mathrm{Fnol}}Z_{\mathrm{lk}}+\overset{1}{\underset{l=0}{\sum }}a_{\mathrm{Mol}}Z_{\mathrm{lk}}+\overset{2}{\underset{l=0}{\sum }}pe_{\mathrm{Mol}}Z_{\mathrm{lk}}+e_{\mathrm{ijkno}},$$

where $y_{\mathrm{ijkno}}$ is a vector of repeated biweekly records for either FERT or HOF or HOS of the *n*th hen mated to the *o*th tom at the *k*th age belonging to the *i*th hatch week, *µ* is the overall mean, $hw_{i}$ is a vector of the fixed effect of the *i*th hatch week, $\mathrm{eh}w_{j}$ is a vector of the fixed effect of the *j*th egg hatch week, $b_{l}$ is a fixed regression coefficient of age of the hen when records were collected, $X_{\mathrm{lk}}$ is the incidence matrix value of the *l*th degree Legendre polynomial fitted for the effect of *k*th age, $a_{\mathrm{Fnol}}$ is the RR coefficient of additive genetic effect of the *n*th hen mated to the *o*th tom, $a_{\mathrm{Mol}}$ is the RR of additive genetic effect of the *o*th tom, $pe_{\mathrm{Fnol}}$ is the RR coefficient of permanent environment effect of the hen mated to the *o*th tom, $pe_{\mathrm{Mol}}$ is the RR coefficient of permanent environment effect of the *o*th tom, $Z_{\mathrm{lk}}$ is the incidence matrix value of the *l*th degree Legendre polynomial fitted for the additive genetic and permanent environment effects at *k*th age, and $e_{\mathrm{ijkno}}$ is the residual error term. The assumptions of the random effects were: $a_{Fno}∼N\left(0,\;A\sigma_{a_{Fno}}^{2}\right)$ , $a_{Mo}∼N\left(0,A\sigma_{a_{Mo}}^{2}\right)$ , $pe_{Mo}∼N\left(0,\;I\sigma_{pe_{Mo}}^{2}\right)$ , $pe_{Fno}∼N\left(0,\;I\sigma_{pe_{Fno}}^{2}\right)$ , and $e_{ijkno}∼N\left(0,\;I\sigma_{e_{ijkno}}^{2}\right)$ , where $\sigma_{a_{Fno}}^{2}$ is the hen additive genetic variance, $\sigma_{a_{Mo}}^{2}$ is the tom additive genetic variance, $\sigma_{pe_{Mo}}^{2}$ is the tom permanent environment variance, $\sigma_{pe_{Fno}}^{2}$ is the hen permanent environment variance, $\sigma_{e_{ijkno}}^{2}$ is the error variance, and A is the numerator relationship matrix. Also, the model was fitted using heterogeneous residuals per age class. For the purpose of comparison between CUM and RR models, variance components estimated for biweekly ages with the RR model were averaged to produce a single value for the additive genetic variance, permanent environment variance, and repeatability.

### Single-step genomic best linear unbiased prediction

Following the presentation of creating a relationship matrix that included pedigree and genomic information (Legarra et al., 2009), an H relationship matrix derived from the combination of pedigree and genomic data was created to replace the pedigree relationship matrix (A) used in the aforementioned animal mixed model. Due to the computational cost of computing the H matrix, the inverse of H matrix is computed with a simpler structure:

$$H^{-1}=A^{-1}+\left[\begin{array}{cc} 0 & 0\\ 0 & {\left(0.95G+0.05A_{22}\right)}^{-1}-A_{22}^{-1} \end{array}\right],$$

where $A^{-1}$ is the inverse of the pedigree relationship matrix, $A_{22}^{-1}$ is the inverse of the **A** matrix of only the genotyped animals, and $G^{-1}$ is the inverse of the genomic relationship matrix estimated using the method presented by VanRaden (2008). Singular matrices are not invertible; therefore, to ensure that the G matrix is invertible, 0.05 of $A_{22}$ was added to 0.95 of G. These weighting parameters were chosen because Abdalla et al. (2019) observed slightly more improvement in breast meat yield in turkeys with these weightings.

Estimates of the RR coefficients from BLUP and ssGBLUP RR models were used to derive the pedigree-estimated breeding value (EBV) and genomic-estimated breeding value (GEBV), respectively.

$$EBV_{i}=W{\hat{\alpha }}_{i},$$

$$GEBV_{i}=W{\hat{\delta }}_{i},$$

where ${\hat{\alpha }}_{i}$ is the estimated additive genetic regression coefficients for the *i*th animal, ${\hat{\delta }}_{i}$ is the estimated additive genomic regression coefficients for the *i*th animal, and ***W*** is a matrix of age covariate ranging from 38 to 62 weeks and associated with the degree of Legendre polynomials used.

### Variance components and model comparison

**FIGURE 1:**
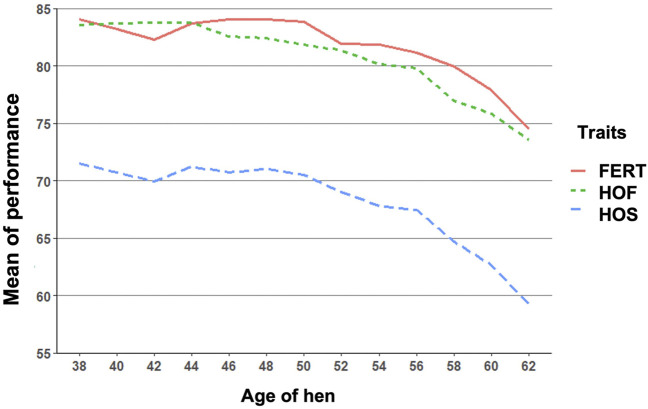
Biweekly mean of fertility of set (FERT), hatch of fertile (HOF), and hatch of set (HOS) from 38 to 62 weeks of hen age when records were measured.

All variance components and genetic parameters used in this study were estimated using the WOMBAT software program (Meyer, 2007). To adequately capture the parameters that contribute to the variation observed in reproductive traits when using RR models, three different models with varying degrees of Legendre polynomials were compared as shown in Table 3. The first model was a full model that had the highest possible degree of Legendre polynomials that converged. The second and third models were reduced models with lower degrees of Legendre polynomials. The criteria used to choose the best model were based on the log likelihood ratio test, the most parsimonious model that converged with both pedigree and genomic information and the Akaike information criterion (Akaike, 1998). For the fixed effect, a cubic polynomial was used because it appropriately described the trend in the biweekly reproductive traits as shown in Figure 1.

**TABLE 3:** Summary of the fitted degree of Legendre polynomials^a^ used in the random regression model with their corresponding log likelihoods (logL), Akaike information criteria (AIC), and the significance of their log likelihood ratio test (LRT) relative to the full model.

Model	$a_{F}$	$a_{M}$	$pe_{F}$	$pe_{M}$	logL	AIC	LRT
1	3	2	3	2	-231144.99	462382	Full model
2^b^	3	1	3	2	-231145.36	462376.71	NS
3	3	1	3	1	-231183.22	462446.43	^c^

*a*_F_ = additive genetic effect of the hen; *a*_M_ = additive genetic effect of the tom; *pe*_F_ = permanent environment effect of the hen; *pe*_M_ = permanent environment effect of the tom.

a Degree of Legendre polynomials: 1 = linear regression; 2 = quadratic regression; 3 = cubic regression.

b Model with the best fit in comparison to the full model.

c *p* value <0.001; NS: no significant difference; full model: model that had the highest number of parameters indicated by higher degree of Legendre polynomials.

### Predictive ability

Predictive ability of BLUP and ssGBLUP for all models was assessed using the following technique. Initially, observed phenotypes were corrected for all fixed effects fitted in the full model based on traditional BLUP (adjusted phenotype). Next, phenotypic records were removed for approximately 10% (the youngest animals) defined by hatch between the year 2018 and 2019 (reduced data set). These young animals were assigned to the validation population, and the remaining 90% animals were assigned to the training population. Thereafter, EBV and GEBV for the validation population were estimated. The predictive ability for each trait was calculated as the Pearson correlation coefficient between the EBV or GEBV estimated based on the reduced data set and the adjusted phenotype estimated from the full data set from the CUM model, while considering only animals in the validation population. Likewise, Pearson correlation coefficients between biweekly adjusted phenotypes from the full model and the biweekly EBV or GEBV estimated from the reduced data set and only considering animals in the validation population were used for the RR model**.**

## Results

### Data structure

The observed mean for FERT, HOF, and HOS was 81.27, 80.86, and 67.94%, respectively, for single records used for the CUM model as shown in Table 1. Biweekly averages of FERT, HOF, and HOS records collected during the productive life of the hen are plotted in Figure 1 and presented in Table 2. The reproductive performance trend shows an average of approximately 84.0% at the early stages of the hen’s productive life, with a noticeable decline at the later stages of production/lay, decreasing to approximately 75.0% for FERT and HOF. Similarly, HOS was high at the beginning of production with an initial value of approximately 71.0%; however, after week 52, a steady decrease was observed until 62 weeks with a value of 59.0%.

### Genetic parameters of different models with pedigree and genomic information

Estimates of the variance components for all the models implemented are presented in Table 4. For the CUM model using only pedigree information, the additive genetic variances ranged from 16.50 to 55.05 for all three traits; however, the inclusion of genomic information consequently increased the additive genetic variance by approximately 36, 60, and 36% for FERT, HOF, and HOS, respectively. Generally, the average additive genetic variances estimated using RR models were higher than those estimated using CUM and ranged from 35.86 to 108.98 for all traits with only pedigree information. With the addition of genomic information, average additive genetic variance increased to 45.06, 137.08, and 125.46 for FERT, HOF, and HOS, respectively. The residual variances from the CUM model were on average 5% lower for all traits when using pedigree and genomic information than using only pedigree information. In contrast, for the RR model, there were no substantive differences in the estimated residual variances between pedigree only and with the addition of genomic information.

**TABLE 4:** Estimates of additive, permanent, error, and phenotypic variances, heritability, and repeatability.

Trait	Model	Relationship^a^	$\sigma_{a}^{2}$	$\sigma_{\mathrm{pe}}^{2}$	$\sigma_{e}^{2}$	$\sigma_{p}^{2}$	$h^{2}$	re
FERT	Cumulative	P	16.50	—	128.92	145.42	0.11 ± 0.02	—
		P and G	22.27	—	126.36	148.63	0.15 ± 0.02	—
	Random regression^b^	P	35.86	86.79	296.83	635.42	0.06 ± 0.01	0.19
		P and G	45.06	84.05	296.77	638.94	0.08 ± 0.01	0.20
HOF	Cumulative	P	49.21	—	157.27	206.48	0.24 ± 0.02	—
		P and G	78.67	—	144.68	223.35	0.35 ± 0.02	—
	Random regression^b^	P	108.98	117.82	477.88	799.77	0.14 ± 0.02	0.29
		P and G	137.08	109.58	478.28	815.70	0.17 ± 0.02	0.31
HOS	Cumulative	P	55.05	—	179.32	234.37	0.24 ± 0.02	—
		P and G	75.00	—	171.71	246.72	0.30 ± 0.02	—
	Random regression^b^	P	102.43	144.98	435.05	810.46	0.14 ± 0.02	0.31
		P and G	125.46	138.85	435.33	822.42	0.16 ± 0.02	0.32

a P, pedigree information only; P & G, pedigree and genomic information.

b All variance components, heritability, and repeatability for the random regression are average estimates across time point.

**FIGURE 2:**
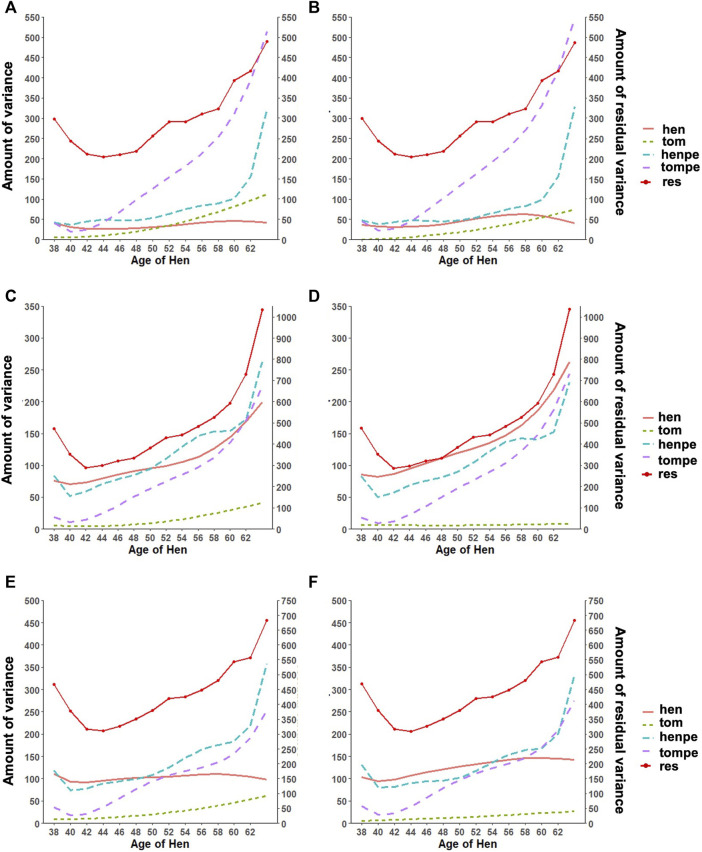
Estimates of variance components for **(A)** fertility (FERT) with pedigree, **(B)** FERT with genotypes, **(C)** hatch of fertile (HOF) with pedigree, **(D)** HOF with ssGBLUP, **(E)** hatch of set (HOS) with pedigree, and **(F)** HOS with ssGBLUP from the random regression model with linear regression for the additive genetic variance of the hen (hen) and tom (tom), the permanent environment variance of the hen (henpe) and tom (tompe), and the residual variance (res).

An important reason for the implementation of RR is the ability to appropriately model the trajectory of longitudinal traits. This accounts for both the additive and permanent environment effect for traits with repeated records. The trends observed in the variance components estimated with the RR model are shown in Figure 2. The estimated additive genetic variance for HOF shows that the hen predominantly contributes to the observed variation, with close to zero contribution from the sire. A similar pattern was found with HOS; however, the sire contribution to the additive genetic variation increased steadily toward the end of production with a slight decline observed in the genetic variation of the hen. For FERT, both the hen and the sire contributed considerably to the additive genetic variance, with the sire having less contribution at the early stages and more contribution at the later stages of production. The permanent environment variances of the sire and hen increased gradually from the beginning of production to the end of production for all traits except for the permanent environment variance of the hen for FERT, which was constant throughout the production with a steep increase at the later production stages. In general, the addition of genomic information resulted in an increase in the additive genetic variances contributed by the hen for all traits with almost no changes in the other variance components.

**FIGURE 3:**
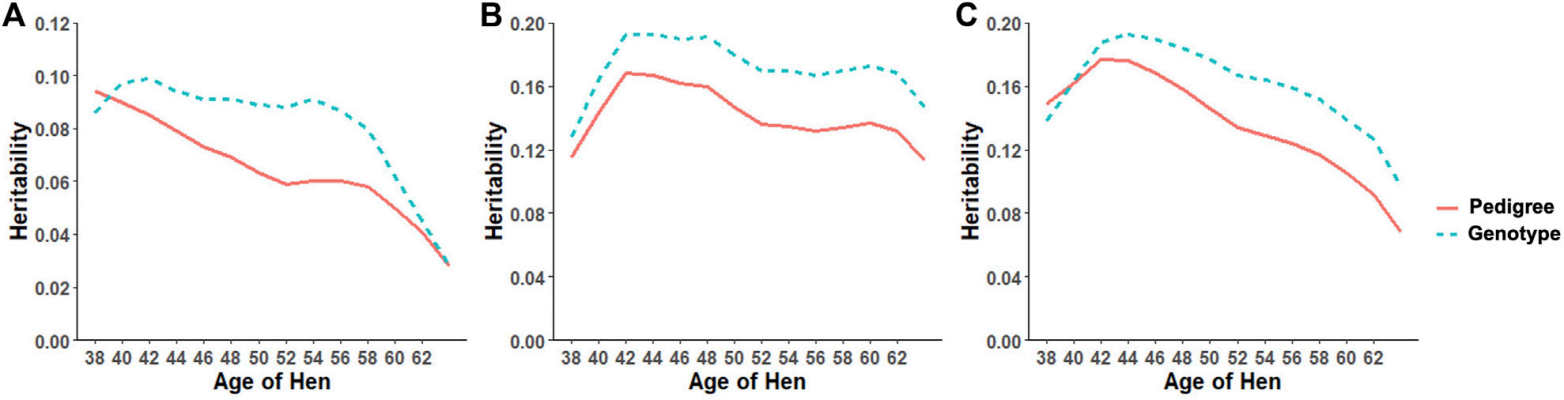
Heritability trend for different age classes for **(A)** fertility (FERT), **(B)** hatch of fertile (HOF), and **(C)** hatch of set (HOS) estimated from the random regression model using pedigree and genotypic information.

Heritability estimates from the CUM model for the traits ranged from 0.11 to 0.24 for all traits with pedigree information (Tables 5, 6, 7, 8, 9, 10). On average, heritability estimates increased by approximately 28% for all traits with the addition of genomic information. Although not directly comparable to the CUM model, the average heritability estimates from the RR model with pedigree and genomic information was estimated to be 0.08, 0.17, and 0.16 for FERT, HOF, and HOS, respectively. Pedigree estimates of heritability from the RR model were an average of 23% lower than estimates with the inclusion of genomic information. The trend in heritability estimated from the RR model ranged from 0.04 to 0.10, 0.11 to 0.19, and 0.09 to 0.19 for FERT, HOF, and HOS, respectively (Figure 3). The peak for heritability estimates was observed at 42 weeks of age or from eggs produced approximately 1 month into production for all traits, and the lowest estimates were found at the end of production for FERT and HOS and 2 weeks into production for HOF. Overall, heritability estimates from pedigree and genomic information were higher than pedigree estimates.

**TABLE 5:** Estimates of heritability (diagonal), phenotypic correlations (below diagonal), and genetic correlations (above diagonal) for fertility (FERT) using the pedigree random regression model.

Age	38	40	42	44	46	48	50	52	54	56	58	60	62
38	0.09 ± 0.02	0.97	0.91	0.86	0.82	0.81	0.81	0.81	0.79	0.78	0.76	0.72	0.66
40	0.25	0.09 ± 0.01	0.98	0.95	0.92	0.90	0.88	0.86	0.83	0.79	0.76	0.71	0.65
42	0.19	0.27	0.09 ± 0.01	0.99	0.97	0.96	0.93	0.89	0.84	0.80	0.75	0.69	0.64
44	0.14	0.25	0.34	0.08 ± 0.01	0.99	0.98	0.95	0.91	0.86	0.81	0.76	0.71	0.65
46	0.10	0.23	0.33	0.39	0.07 ± 0.01	0.99	0.97	0.94	0.89	0.84	0.79	0.75	0.69
48	0.08	0.20	0.31	0.39	0.44	0.07 ± 0.01	0.99	0.97	0.93	0.89	0.84	0.80	0.75
50	0.07	0.17	0.28	0.36	0.42	0.46	0.06 ± 0.01	0.99	0.97	0.93	0.90	0.86	0.81
52	0.06	0.15	0.25	0.33	0.40	0.46	0.48	0.06 ± 0.01	0.99	0.97	0.94	0.92	0.87
54	0.05	0.14	0.23	0.32	0.39	0.45	0.49	0.51	0.06 ± 0.01	0.99	0.98	0.95	0.92
56	0.05	0.12	0.21	0.29	0.37	0.43	0.47	0.50	0.54	0.06 ± 0.01	0.99	0.98	0.95
58	0.04	0.11	0.19	0.27	0.35	0.41	0.46	0.49	0.53	0.56	0.06 ± 0.01	0.99	0.97
60	0.03	0.10	0.18	0.25	0.31	0.37	0.41	0.44	0.49	0.53	0.57	0.05 ± 0.01	0.99
62	0.02	0.09	0.17	0.23	0.28	0.33	0.36	0.39	0.44	0.49	0.54	0.58	0.04 ± 0.01

**TABLE 6:** Estimates of heritability (diagonal), phenotypic correlations (below diagonal), and genetic correlations (above diagonal) for fertility (FERT) using the ssGBLUP random regression model.

Age	38	40	42	44	46	48	50	52	54	56	58	60	62
38	0.09 ± 0.02	0.99	0.98	0.97	0.95	0.92	0.88	0.85	0.83	0.81	0.81	0.83	0.87
40	0.26	0.10 ± 0.01	0.99	0.98	0.96	0.92	0.88	0.83	0.80	0.78	0.78	0.79	0.82
42	0.20	0.28	0.10 ± 0.01	0.99	0.97	0.94	0.89	0.85	0.82	0.79	0.78	0.79	0.80
44	0.15	0.26	0.34	0.09 ± 0.01	0.99	0.96	0.93	0.89	0.86	0.84	0.82	0.82	0.82
46	0.12	0.23	0.34	0.40	0.09 ± 0.01	0.99	0.97	0.94	0.92	0.89	0.87	0.86	0.85
48	0.09	0.21	0.32	0.39	0.44	0.09 ± 0.01	0.99	0.97	0.96	0.94	0.92	0.91	0.88
50	0.08	0.18	0.29	0.37	0.43	0.47	0.09 ± 0.01	0.99	0.98	0.97	0.95	0.94	0.90
52	0.07	0.16	0.26	0.34	0.41	0.46	0.49	0.09 ± 0.01	0.99	0.99	0.98	0.96	0.92
54	0.06	0.15	0.24	0.32	0.40	0.46	0.49	0.52	0.09 ± 0.01	0.99	0.99	0.97	0.93
56	0.06	0.13	0.22	0.30	0.37	0.44	0.48	0.51	0.55	0.09 ± 0.01	0.99	0.98	0.95
58	0.05	0.12	0.20	0.28	0.35	0.42	0.46	0.50	0.54	0.56	0.08 ± 0.01	0.99	0.97
60	0.04	0.11	0.18	0.25	0.32	0.37	0.42	0.45	0.49	0.53	0.57	0.06 ± 0.01	0.98
62	0.02	0.10	0.17	0.23	0.28	0.33	0.36	0.39	0.44	0.49	0.54	0.58	0.05 ± 0.01

**TABLE 7:** Estimates of heritability (diagonal), phenotypic correlations (below diagonal), and genetic correlations (above diagonal) for hatch of fertile (HOF) using the pedigree random regression model.

Age	38	40	42	44	46	48	50	52	54	56	58	60	62
38	0.12 ± 0.02	0.96	0.86	0.77	0.69	0.64	0.60	0.58	0.57	0.57	0.58	0.59	0.61
40	0.25	0.14 ± 0.02	0.97	0.921	0.869	0.825	0.790	0.761	0.74	0.71	0.69	0.68	0.67
42	0.19	0.29	0.17 ± 0.02	0.98	0.95	0.92	0.89	0.87	0.83	0.79	0.76	0.73	0.70
44	0.15	0.26	0.35	0.17 ± 0.02	0.99	0.97	0.953	0.92	0.88	0.85	0.80	0.76	0.72
46	0.12	0.24	0.34	0.38	0.16 ± 0.02	0.99	0.98	0.95	0.93	0.88	0.84	0.79	0.74
48	0.10	0.22	0.32	0.37	0.39	0.16 ± 0.02	0.99	0.98	0.95	0.92	0.87	0.82	0.78
50	0.09	0.19	0.29	0.34	0.38	0.40	0.15 ± 0.01	0.99	0.98	0.95	0.91	0.86	0.82
52	0.09	0.17	0.26	0.31	0.35	0.39	0.40	0.14 ± 0.01	0.99	0.98	0.94	0.91	0.87
54	0.09	0.16	0.24	0.29	0.33	0.37	0.39	0.41	0.14 ± 0.02	0.99	0.97	0.95	0.91
56	0.10	0.15	0.21	0.26	0.31	0.35	0.38	0.41	0.43	0.13 ± 0.02	0.99	0.98	0.95
58	0.11	0.15	0.20	0.24	0.28	0.33	0.36	0.39	0.42	0.43	0.13 ± 0.02	0.99	0.98
60	0.11	0.15	0.19	0.23	0.26	0.30	0.33	0.36	0.39	0.42	0.43	0.14 ± 0.02	0.99
62	0.11	0.15	0.19	0.21	0.24	0.27	0.29	0.31	0.34	0.37	0.39	0.42	0.13 ± 0.03

**TABLE 8:** Estimates of heritability (diagonal), phenotypic correlations (below diagonal), and genetic correlations (above diagonal) for hatch of fertile (HOF) using the ssGBLUP random regression model.

Age	38	40	42	44	46	48	50	52	54	56	58	60	62
38	0.13 ± 0.02	0.96	0.84	0.80	0.73	0.68	0.65	0.63	0.63	0.64	0.67	0.69	0.73
40	0.257	0.16 ± 0.02	0.98	0.93	0.88	0.84	0.81	0.79	0.78	0.77	0.77	0.77	0.78
42	0.212	0.30	0.19 ± 0.02	0.99	0.96	0.93	0.91	0.89	0.87	0.85	0.83	0.81	0.79
44	0.163	0.28	0.36	0.19 ± 0.02	0.99	0.98	0.96	0.94	0.92	0.89	0.86	0.83	0.79
46	0.131	0.26	0.35	0.39	0.19 ± 0.02	0.99	0.98	0.97	0.95	0.92	0.88	0.84	0.80
48	0.115	0.24	0.33	0.39	0.41	0.19 ± 0.02	0.99	0.98	0.97	0.94	0.91	0.86	0.82
50	0.106	0.21	0.30	0.36	0.39	0.42	0.18 ± 0.01	0.99	0.98	0.96	0.93	0.89	0.85
52	0.104	0.19	0.27	0.33	0.37	0.40	0.42	0.17 ± 0.01	0.99	0.98	0.96	0.92	0.88
54	0.110	0.18	0.25	0.30	0.35	0.39	0.41	0.43	0.17 ± 0.02	0.99	0.98	0.95	0.92
56	0.116	0.17	0.23	0.28	0.32	0.37	0.39	0.42	0.44	0.17 ± 0.02	0.99	0.98	0.95
58	0.122	0.17	0.22	0.26	0.30	0.34	0.37	0.40	0.43	0.45	0.17 ± 0.02	0.99	0.98
60	0.126	0.17	0.21	0.25	0.28	0.32	0.34	0.37	0.41	0.43	0.44	0.17 ± 0.02	0.99
62	0.122	0.17	0.21	0.23	0.25	0.28	0.30	0.32	0.35	0.38	0.41	0.43	0.17 ± 0.02

**TABLE 9:** Estimates of heritability (diagonal), phenotypic correlations (below diagonal), and genetic correlations (above diagonal) for hatch of set (HOS) using the pedigree random regression model.

Age	38	40	42	44	46	48	50	52	54	56	58	60	62
38	0.15 ± 0.02	0.95	0.84	0.74	0.67	0.63	0.60	0.58	0.57	0.56	0.56	0.55	0.55
40	0.31	0.16 ± 0.02	0.97	0.91	0.86	0.82	0.78	0.75	0.71	0.68	0.66	0.65	0.65
42	0.25	0.33	0.18 ± 0.02	0.98	0.95	0.92	0.88	0.84	0.80	0.75	0.72	0.70	0.70
44	0.19	0.30	0.39	0.18 ± 0.02	0.99	0.97	0.94	0.90	0.86	0.80	0.77	0.74	0.75
46	0.15	0.28	0.37	0.42	0.17 ± 0.02	0.99	0.97	0.94	0.90	0.85	0.81	0.79	0.79
48	0.13	0.25	0.35	0.41	0.44	0.16 ± 0.01	0.99	0.97	0.93	0.90	0.86	0.84	0.84
50	0.11	0.22	0.32	0.38	0.42	0.45	0.15 ± 0.01	0.99	0.97	0.94	0.91	0.89	0.89
52	0.11	0.20	0.29	0.35	0.39	0.43	0.45	0.13 ± 0.01	0.99	0.97	0.95	0.94	0.93
54	0.12	0.19	0.26	0.32	0.37	0.41	0.44	0.46	0.13 ± 0.01	0.99	0.98	0.97	0.96
56	0.12	0.18	0.24	0.29	0.34	0.39	0.43	0.45	0.48	0.12 ± 0.02	0.99	0.98	0.97
58	0.12	0.17	0.22	0.27	0.32	0.36	0.40	0.43	0.46	0.48	0.12 ± 0.02	0.99	0.98
60	0.12	0.16	0.21	0.25	0.29	0.33	0.36	0.39	0.43	0.45	0.47	0.11 ± 0.02	0.99
62	0.11	0.16	0.20	0.24	0.27	0.29	0.32	0.34	0.38	0.41	0.45	0.47	0.09 ± 0.02

**TABLE 10:** Estimates of heritability (diagonal), phenotypic correlations (below diagonal), and genetic correlations (above diagonal) for hatch of set (HOS) using the ssGBLUP random regression model.

Age	38	40	42	44	46	48	50	52	54	56	58	60	62
38	0.14 ± 0.02	0.95	0.87	0.79	0.74	0.70	0.68	0.66	0.65	0.65	0.66	0.67	0.68
40	0.32	0.16 ± 0.02	0.97	0.93	0.89	0.85	0.83	0.80	0.78	0.76	0.74	0.74	0.74
42	0.26	0.34	0.19 ± 0.02	0.98	0.96	0.94	0.91	0.88	0.85	0.82	0,79	0.78	0.78
44	0.19	0.31	0.40	0.19 ± 0.02	0.99	0.97	0.95	0.92	0.89	0.86	0.83	0.81	0.80
46	0.16	0.29	0.39	0.44	0.19 ± 0.02	0.99	0.980	0.95	0.92	0.89	0.87	0.85	0.83
48	0.14	0.26	0.37	0.42	0.45	0.18 ± 0.01	0.99	0.98	0.95	0.93	0.90	0.88	0.86
50	0.13	0.24	0.33	0.40	0.43	0.46	0.18 ± 0.01	0.99	0.98	0.96	0.93	0.91	0.89
52	0.12	0.21	0.30	0.36	0.41	0.44	0.46	0.17 ± 0.01	0.99	0.98	0.96	0.94	0.92
54	0.13	0.20	0.28	0.33	0.38	0.43	0.46	0.48	0.16 ± 0.02	0.99	0.98	0.97	0.95
56	0.13	0.19	0.26	0.31	0.35	0.40	0.44	0.47	0.49	0.16 ± 0.02	0.99	0.98	0.96
58	0.14	0.18	0.23	0.28	0.33	0.37	0.41	0.44	0.47	0.49	0.15 ± 0.02	0.99	0.98
60	0.13	0.18	0.22	0.26	0.30	0.34	0.37	0.40	0.43	0.46	0.48	0.14 ± 0.02	0.99
62	0.12	0.17	0.22	0.25	0.28	0.30	0.33	0.36	0.39	0.41	0.45	0.48	0.13 ± 0.02

Phenotypic and genetic correlations from the RR model are shown in Tables 5, 6, 7, 8, 9, 10. Estimated genetic correlation was found to be very high for adjacent weeks and ranged from 0.96 to 0.99. However, as the distance between the weeks increased, the correlations declined and varied from 0.57 to 0.98. In a similar pattern, phenotypic correlations were higher for closer weeks than for weeks further apart. Phenotypic correlations were substantially lower than genetic correlations and ranged from 0.02 to 0.58.

### Predictive ability

**FIGURE 4:**
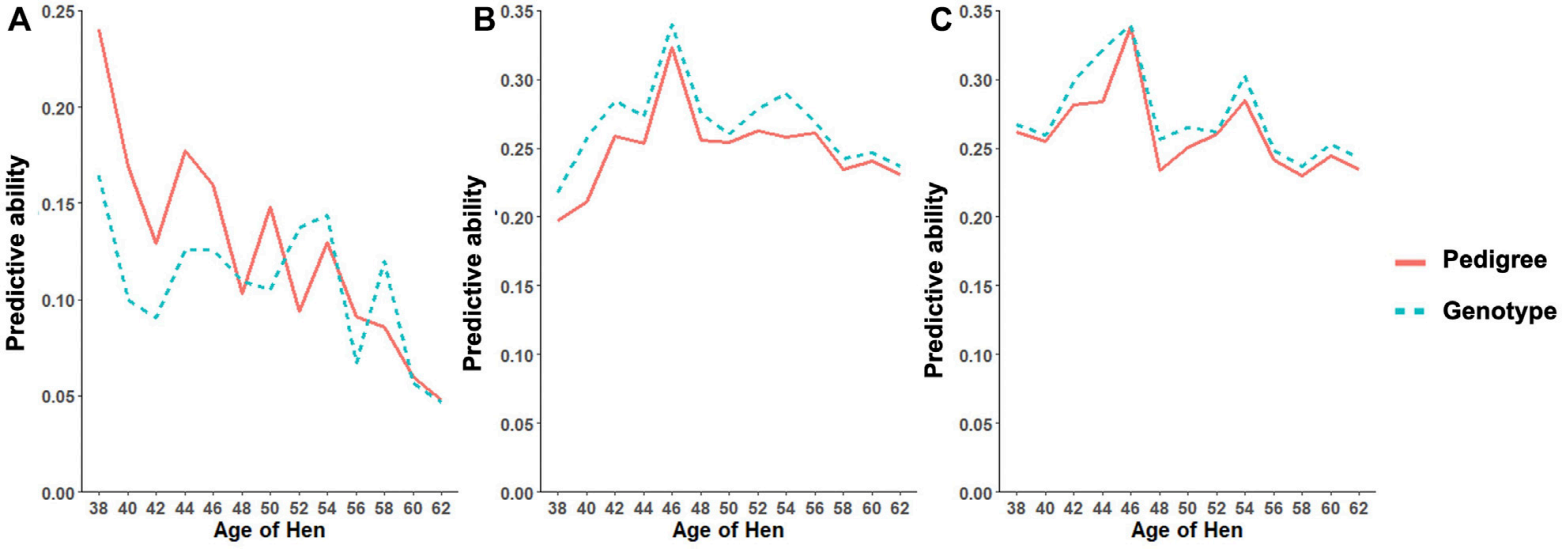
Predictive ability trend for different age classes for **(A)** fertility (FERT), **(B)** hatch of fertile (HOF), and **(C)** hatch of set (HOS) estimated from the random regression model using pedigree and genotypic information.

As shown in Table 11, the predictive ability of BLUP and ssGBLUP was assessed for all models considered in this study. Across most of the studied traits and models, BLUP was outperformed by ssGBLUP with higher predictivity, and only FERT showed lower ssGBLUP predictivity than BLUP. For the CUM model, BLUP predictive ability was estimated to be 0.23, 0.22, and 0.23 for FERT, HOF, and HOS, respectively. With the incorporation of genomics, these predictivities increased by 14 and 9% for HOF and HOS, respectively, and reduced by 9% for FERT. To facilitate comparison with the CUM model, average predictive ability were estimated for the RR model, which ranged from 0.11 to 0.27 for all studied traits. The biweekly trend of the predictivities estimated from the RR model is shown in Figure 4. The figure shows the maximum predictive ability estimated at approximately 44–46 weeks of age for HOF and HOS using BLUP and ssGBLUP. Conversely, the maximum predictivity for FERT was observed at 38 weeks. Predictivities estimated from BLUP were generally lower than those estimated from ssGBLUP across all biweekly records and for all traits. However, BLUP predictive ability at the early stages of production was higher than ssGBLUP for FERT.

**TABLE 11:** Estimates of predictive ability using cumulative and random regression models with pedigree and genomic information.

Trait	Model	Relationship^a^	Predictive ability
FERT	Cumulative	P	0.23
		P and G	0.21
	Random regression	P	0.13
		P and G	0.11
HOF	Cumulative	P	0.22
		P and G	0.25
	Random regression	P	0.25
		P and G	0.27
HOS	Cumulative	P	0.23
		P and G	0.25
	Random regression	P	0.26
		P and G	0.27

a P, pedigree information only; P and G, pedigree and genomic information.

## Discussion

The present study sought to estimate genetic parameters for reproductive traits in a maternal turkey line using CUM and RR models with pedigree and genomic information. Estimated average HOF and FERT were approximately 80%, which is close to the estimates reported by Case et al. (2010). The slightly lower estimates from this study could be attributable to differences in the population used. Presently, there are no literature reports on HOS and limited reports on FERT and HOF in turkeys; hence, results from layer and broiler chickens were used for comparison. The mean HOS in this study was 18% lower than HOF, which is within the range reported for chickens (Wolc et al., 2010; Wolc et al., 2019). In accordance with previous studies, the trajectory trend in biweekly FERT, HOF, and HOS are similar to those reported in turkeys (Dunnington et al., 1990), in broilers (Heier and Jarp, 2001; Wolc et al., 2009), and in layers (Wolc et al., 2007). This trend supports the characteristically declining feature of the traits over the productive life of the hen as well as the longitudinal nature of these traits.

### Genetic parameters between different models with pedigree and genomic information

The present study shows that the additive genetic effect of both the hen and sire plays a significant role in the observed variation in FERT, which is in line with the study published by Wolc et al. (2009) in broiler chickens with natural mating. Conversely, the sire additive genetic effect had a non-significant contribution to the variation observed in HOF. Similar results were reported in broiler chickens (Wolc et al., 2010), which may be due to the limited effect of the male after fertilization and more pronounced effect of the hen based on the environment provided for the developing embryo (quality of egg produced by the hen) (Wolc and Olori, 2009). Estimated additive genetic and error variances were smaller for all studied traits with the CUM model than with the RR model. The reduced variances may be because of accumulating repeated records as a single record per animal, thereby removing the covariance that exists between repeated records. With the simultaneous combination of pedigree and genomic information, additive genetic variances were higher for all the models and traits than pedigree only information. This outcome demonstrates that genomic information better captures actual relationships between individuals than the expected relationship captured by the pedigree (Hayes and Goddard, 2008). Based on the pedigree, heritability estimates for all traits ranged from 0.06 to 0.24 for all models. These estimates are within the range of 0.08–0.18 reported by Case et al. (2010). As expected, estimated heritabilities from the CUM model were higher than those from the RR model (Anang et al., 2000). This could be due to the reduced residual variance, as well as the inability to account for the correlated structure of the repeated records from the different ages. In addition, some components of the permanent environment variance could be attributed to the additive genetic variance, which would not be easily removed due to the cumulation of records. From the RR model, heritability increased from 38 weeks of age when records were measured to a maximum at approximately week 42–46 and varied across all productive ages. Similar trends across multiple time points have been found for FERT, HOF, and egg production in turkeys (Kranis et al., 2007; Case, 2011). This indicates that RR properly accounts for environmental differences throughout the productive life of the animals. This may also indicate that different genes are being expressed at different times across the productive life of the animals. Overall, heritability estimated using the combination of pedigree and genomic relationship for all traits and models was higher than that using only pedigree relationships. Higher estimates of heritability with ssGBLUP than BLUP have also been reported in turkeys for feed conversion ratio, residual feed intake, body weight, breast meat yield, and walking score (Abdalla et al., 2019). Correlations estimated in this study showed that proximate ages had higher correlations, which declined as the ages became further apart. This pattern is similar to studies from test-day milk yield in dairy cattle (Jamrozik and Schaeffer, 1997) and goat (Brito et al., 2017). This could indicate that repeated records of the same trait collected at different ages represent different traits, especially when the time points are further apart.

### Predictive ability

Predictive ability estimated based on ssGBLUP had higher estimates than BLUP for most traits. This is expected as many studies have shown lower predictivity from pedigree-based EBV than marker-based EBV (Hayes et al., 2010; Abdalla et al., 2019; Oliveira et al., 2019). Similarly, Lourenco et al. (2015) reported an increase in ssGBLUP predictive ability that ranged from 0.05 to 0.1 for birth weight in beef cattle relative to BLUP. In contrast to the higher predictions of marker-based EBV, pedigree-based EBV prediction was higher than marker-based prediction for FERT in this study. Wolc et al. (2019) found similar results for male fertility in White Leghorns layers with natural floor pen mating. This may be because fertility is influenced largely by environmental factors, and in this study, FERT had the lowest heritability among all studied traits. Another possibility is that some causal genomic regions could be located on the sex chromosomes, which were removed from these analyses. Furthermore, the CUM model had slightly higher predictability than the RR model. This is also expected because of the higher heritability estimates from the CUM model. However, these higher heritability estimates could be overestimated due to the inability to account for the permanent environment effects observed from longitudinal traits (Anang et al., 2000).

## Conclusion

In this study, the applicability of RR ssGBLUP was investigated and compared to the traditional CUM model used in estimating the reproductive trait in turkeys. Our findings suggest that genomic relationships result in higher heritability estimates over traditional pedigree relationships, consequently causing higher predictive ability. In addition, the RR model captured the covariance and correlation that exist between different ages throughout the productive life of the animal. Therefore, the use of RR with the incorporation of genomic information is a feasible endeavor for analyzing longitudinal traits like FERT, HOF, and HOS in turkeys.

## Data Availability

Data that support the findings of this study are available from Hybrid Turkey upon reasonable request to the corresponding author, but restrictions apply to the availability of these data, which were used under a license of a material transfer agreement for the current study, and thus are not publicly available.
